# Effects of COVID-19 Pandemic Response on Service Provision for Sexually Transmitted Infections, HIV, and Viral Hepatitis, England

**DOI:** 10.3201/eid2803.211998

**Published:** 2022-03

**Authors:** Holly D. Mitchell, Tatiana Garcia Vilaplana, Sema Mandal, Natasha Ratna, Megan Glancy, Ammi Shah, Ruth Simmons, Celia Penman, Freja Kirsebom, Annastella Costella, Alison E. Brown, Hamish Mohammed, Valerie Delpech, Katy Sinka, Gwenda Hughes, on behalf of the UK Health Security Agency National STI, HIV and Viral Hepatitis Surveillance Group

**Affiliations:** UK Health Security Agency, London, UK

**Keywords:** COVID-19, sexually transmitted infections, coronavirus disease, severe acute respiratory syndrome coronavirus 2, SARS-CoV-2, HIV, HIV/AIDS and other retroviruses, viral hepatitis, health disparities, healthcare services, communicable diseases, viruses, England

## Abstract

Since the coronavirus disease pandemic response began in March 2020, tests, vaccinations, diagnoses, and treatment initiations for sexual health, HIV, and viral hepatitis in England have declined. The shift towards online and outreach services happened rapidly during 2020 and highlights the need to evaluate the effects of these strategies on health inequalities.

Beginning March 23, 2020, the UK government introduced social and physical distancing (SPD) measures to reduce transmission of severe acute respiratory syndrome 2 (SARS-CoV-2), and health staff were redeployed to the coronavirus disease (COVID-19) pandemic response. The staffing shift and SPD measures affected clinical services for sexually transmitted infections (STIs), HIV, and hepatitis A, B, and C (HAV, HBV, and HCV) provided through the National Health Service (*1*,*2*). We assessed the effects of COVID-19 measures in England on service provision in these areas and on health outcomes for persons with STIs, HIV, or hepatitis.

## The Study

In England, surveillance of STIs, HIV, and hepatitis relies on patient-level data on consultations, tests, diagnoses, vaccinations, treatment, and outcomes from sexual health services (SHS), general practitioners, hospital outpatient clinics, and drug treatment centers (*3*). Laboratories also submit patient-level reports of tests and diagnoses for hepatitis and chlamydia. Given the disruption in routine reporting in 2020 (only 71%–98% complete for STI and HIV data), when possible, we analyzed data from clinics and laboratories who provided complete reports for January–September in both 2019 and 2020.

**Figure 1:**
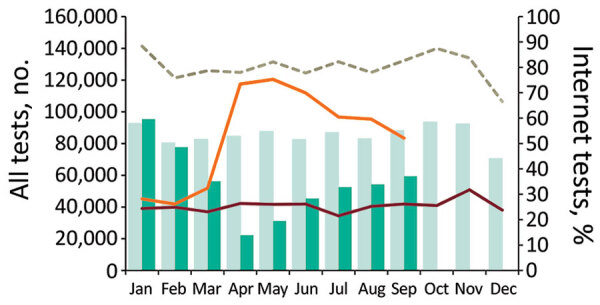
Total number of HIV tests provided through sexual health services (SHS) and proportion of those accessed through internet services, England, January 2019–September 2020. Bars compare HIV test data from SHS that reported complete data for January–September in both 2019 (light green) and 2020 (dark green). Dashed line represents the total number of HIV tests from all SHS reported in each month in 2019. Solid lines indicate the percentages of total tests accessed through the internet for 2019 (red) and 2020 (orange). Data are from routine specialist and nonspecialist SHS reporting to the GUMCAD STI Surveillance System.

Testing at SHS declined by 77%, from 95,455 to 22,332, for HIV and by 71%, from 391,006 to 112,441, for STIs during January–April 2020, and although there was a modest increase beginning in May, testing remained far lower than in 2019 (Figure 1). For January–September 2020 compared with the same period in 2019, overall numbers of tests were lower by 36% (768,216 vs. 494,433) for HIV and 28% (3,137,537 vs. 2,244,153) for STIs (Appendix). However, the proportion of tests accessed through internet services (self-sampling kits returned directly to the laboratory with results provided by text message, email, letter, or online) increased substantially beginning in April 2020 (Appendix). Internet services accounted for ≥63% of HIV and >51% of STI tests during April–September 2020, compared with 25% for HIV and 22% for STIs in 2019.

During January–April 2020, the largest proportional declines in testing occurred among persons 15–19 years of age (79% for HIV, 75% for STIs) and ≥45 years of age (80% for HIV, 76% for STIs); for persons 20–44 years of age, testing for HIV declined by 76% and for STIs by 70%. The 15–19- and >45-year age groups also showed the slowest relative recovery towards prepandemic levels of testing during June–September 2020. Over the same period, we observed larger proportional declines in testing among heterosexual men (81% for HIV, 79% for STIs) and heterosexual or bisexual women (women who have sex with men or women; 76% for HIV, 75% for STIs) compared with gay, bisexual, and other men who have sex with men (MSM) (67% for HIV, 71% for STIs) and lesbian and other women who have sex exclusively with women (66% for HIV, 65% for STIs); recovery was slowest among heterosexual men. We observed the largest declines among persons of Asian (81% for HIV, 77% for STIs), Black (81% for HIV, 76% for STIs), and other (81% for HIV, 76% for STIs) races; persons of Black race showed the slowest recovery. 

We also observed a sharp decline in the number of persons tested for hepatitis during January–April 2020: by 63% (from 4,295 to 1,610) for HAV, 61% (from 57,392 to 22,224) for HBV, and 74% (from 43,238 to 11,250) for HCV (Appendix). The number of persons tested for HCV in community drug treatment facilities showed the greatest decline (98%, from 3,324 to 74) and a slow recovery to prepandemic levels; testing was 58% lower in September 2020 than for 2019 (*3*).

Consistent with testing patterns, the number of diagnoses for HIV, STIs, and hepatitis declined during January–April 2020, followed by a partial recovery (Appendix Figure 1). Bacterial STI positivity (Appendix) increased during March and April 2020 (12% in January 2020 vs. 17% in April 2020) then returned to 2019 and early 2020 levels, whereas HIV test positivity peaked in April 2020 (0.20%) and remained at a higher level until September 2020 (0.09% vs 0.10% in September 2019). By contrast, HBV and HCV positivity declined during January–April 2020 (from 0.8% to 0.4% for HBV surface antigen and from 2.8% to 1.4% for HCV antibody); although there was a slight increase thereafter, positivity remained lower for the rest of 2020 than in 2019.

The number of first-dose vaccinations administered to MSM at SHS during January–April 2020 fell by 97% for HAV (from 841 to 22) and human papillomavirus (from 1,507 to 47) and by 96% (from 757 to 34) for HBV (Appendix Figure 2). A slight increase was reported beginning in May 2020, but rates for HAV, HBV, and HPV vaccinations were >50% lower in September 2020 than in September 2019.

**Figure 2:**
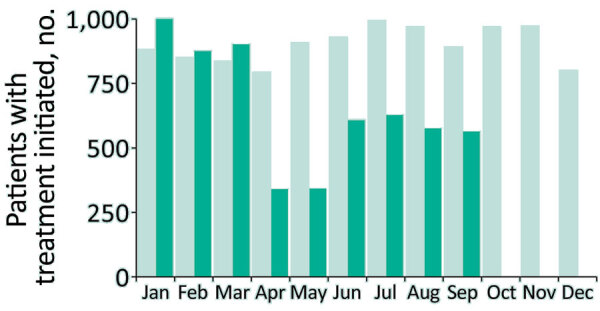
Hepatitis C virus treatment initiations, England, January 2019–September 2020. Data are from the National Health Service England Hepatitis C Patient Registry and Treatment Outcome system. Bars indicate the number of persons having treatment initiated by month for 2019 (light green) and 2020 (dark green).

HCV treatment initiations declined by 66% (from 1,004 to 341) during January–April 2020; although recovering slightly, the overall number of treatment initiations during January–September 2020 was 27% lower than in the same period in 2019 (Figure 2). We saw the largest relative declines for referrals from drug services and prisons, with some recovery during June–September 2020. Delays commonly occur between HCV diagnoses and treatment, so reductions in treatment initiations likely reflected reduced access to services rather than new diagnoses alone.

## Conclusions

The COVID-19 pandemic response in England, including the introduction of SPD measures, coincided with a decline in the provision of, and access to, health services for STIs, HIV, and hepatitis. We observed the greatest decline in services that cannot be provided remotely, such as vaccination.

These findings are supported by staff and peer-support surveys in SHS and community drug treatment services (*4*,*5*). Some reduction in infections and need for services might be a consequence of reduced exposure because of compliance with SPD measures, leading to fewer opportunities for socializing and meeting sexual partners. The partial rebound in the summer of 2020 might indicate some recovery in service provision and demand, with increased demand also influenced by changes in risk perception and behaviors. However, these levels remained below prepandemic levels.

Declines in the numbers of STI, HIV, and hepatitis tests (*6*–*8*) and diagnoses (*8*–*11*) after COVID-19 restrictions began have also been reported across Europe and elsewhere. Disruption in HCV treatment provision is of concern as direct-acting antivirals clear the virus, minimizing long-term harms. HCV treatment disruptions have also been reported in Spain (*12*) and Germany (*13*); in Germany, a minority of treatment providers also reported an increase in delayed diagnoses of liver decompensation and hepatocellular carcinoma (*13*).

During the early stages of the pandemic, there was a rapid shift in service delivery toward online, remote, and outreach provision in England; similar shifts were reported in the United States and Croatia (*14*,*15*). While enabling service access during the pandemic, it will be important to evaluate the effects on health inequalities of changing to remote services, because hepatitis, HIV, and STIs already disproportionately affect socially disadvantaged and excluded groups. Whereas our findings suggest SHS were accessed by some populations of need, such as MSM, the decline in access by young adults and persons of Asian, Black, or other races requires further investigation. Furthermore, early indications of adverse effects on access to harm reduction and bloodborne virus testing services for people who inject drugs is concerning and requires mitigating actions. The full effects of the COVID-19 pandemic response on STI, HIV, and hepatitis infection control, including efforts to eliminate HIV and hepatitis, and longer-term health outcomes will take time to emerge. These effects warrant close monitoring and assessment to ensure services are accessible and used by all who need them.

Appendix Additional information on the effects of COVID-19 pandemic response on providing healthcare for persons with sexually transmitted infections
