# Supplementary material for: Effects of COVID-19 Pandemic Response on Service Provision for Sexually Transmitted Infections, HIV, and Viral Hepatitis, England
Source: Emerg Infect Dis. 2022 Mar;28(3):739–42. doi: 10.3201/eid2803.211998 (PMC8888218; doi:10.3201/eid2803.211998)
Supplement: Appendix — Additional information on the effects of COVID-19 pandemic response on providing healthcare for persons with sexually transmitted infections [file 21-1998-Techapp-s1.pdf]

Article DOI: https://doi.org/10.3201/eid2803.211998

Effects of COVID-19 Pandemic Response on Service Provision for Sexually Transmitted Infections, HIV, and Viral Hepatitis, England

Appendix

STI Testing Data

STI testing data included chlamydia, gonorrhoea, and syphilis, but excluded chlamydia tests in the age group targeted by the National Chlamydia Screening Programme (NCSP). The NCSP provides opportunistic chlamydia screening to sexually active youth 15 to 24 years of age.

Online Testing

Testing for chlamydia and gonorrhoea (either online or in person) is performed according to national guidelines and can include vaginal, rectal, and oral swabs, or a urine sample, depending on individual circumstances. Nucleic acid amplification tests (NAAT) must be used regardless of whether the test was ordered online or taken in-person consultation. Online self-sampling kits for HIV and syphilis require a small blood sample collected from a finger prick, and are typically performed using fourth-generation assays which detect HIV antibodies and p24 antigen simultaneously; syphilis tests typically detect antibodies only. All persons with a reactive result (HIV or syphilis) obtained through online self-sampling are referred to SHSs for further testing and clinical confirmation before a positive diagnosis is reported.

Hepatitis Testing Data

Hepatitis testing data are from sentinel surveillance of bloodborne virus testing in which data are extracted monthly from a network of 16–20 participating laboratories in England. In 2020, sentinel surveillance captured front-line testing covering approximately 40% of the population.

Bacterial STIs

Bacterial STI positivity was calculated using data from all bacterial STI diagnoses including chlamydia, gonorrhoea, *Mycoplasma genitalium*, *Lymphogranuloma venereum* (LGV), nonspecific genital infections, and pelvic inflammatory disease, as a proportion of all screens in which a chlamydia and gonorrhoea test was performed. Infectious syphilis has been excluded because diagnosis usually requires a blood sample.

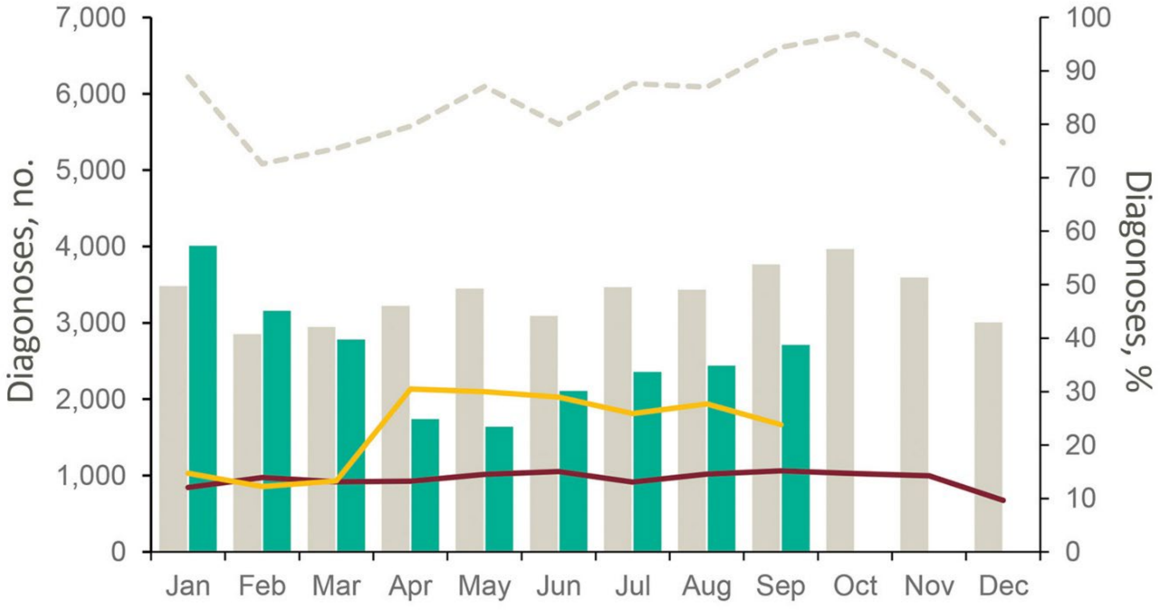

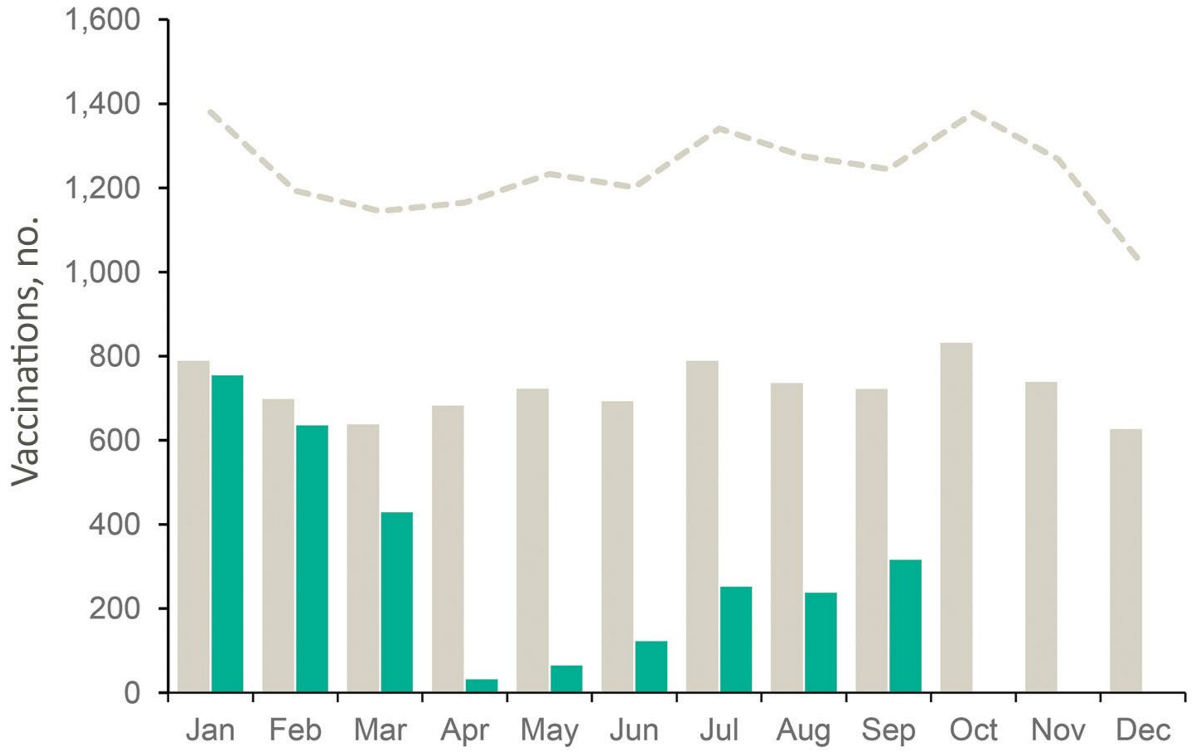

Vaccinations, no.
1,600
1,400
1,200
1,000
800
600
400
200
0
Jan
Feb
Mar
Apr
May
Jun
Jul
Aug
Sep
Oct
Nov
Dec
